# Delaying Reverse Transcription Does Not Increase Sensitivity of HIV-1 to Human TRIM5α

**DOI:** 10.1371/journal.pone.0052434

**Published:** 2013-01-08

**Authors:** Emilie Battivelli, Denise Lecossier, François Clavel, Allan J. Hance

**Affiliations:** 1 Institut National de la Santé et de la Recherche Médicale (INSERM) U941, Paris, France; 2 Institut Universitaire d'Hématologie, Université Paris Diderot, Hôpital Saint-Louis, Paris, France; 3 Service des Maladies Infectieuses et Tropicales, Assistance Publique - Hôpitaux de Paris, Hôpital Bichat - Claude Bernard, Paris, France; Calypte Biomedical Corporation, United States of America

## Abstract

**Background:**

Because uncoating of the capsid is linked to reverse transcription, modifications that delay this process lead to the persistence in the cytoplasm of capsids susceptible to recognition by the human restriction factor TRIM5α (hTRIM5α). It is unknown, however, if increasing the time available for capsid-hTRIM5α interactions would actually render viruses more sensitive to hTRIM5α.

**Results:**

Viral sensitivity to hTRIM5α was evaluated by comparing their replication in human U373-X4 cells in which hTRIM5α activity had or had not been inhibited by overexpression of human TRIM5γ. No differences were observed comparing wild-type HIV-1 and variants carrying mutations in reverse transcriptase or the central polypurine tract that delayed the completion of reverse transcription. In addition, the effect of delaying the onset of reverse transcription for several hours by treating target cells with nevirapine was evaluated using viral isolates with different sensitivities to hTRIM5α. Delaying reverse transcription led to a time-dependent loss in viral infectivity that was increased by inhibiting capsid-cyclophilin A interactions, but did not result in increased viral sensitivity to hTRIM5α, regardless of their intrinsic sensitivity to this restriction factor.

**Conclusions:**

Consistent with prior studies, the HIV-1 capsid can be targeted for destruction by hTRIM5α, but different strains display considerable variability in their sensitivity to this restriction factor. Capsids can also be lost more slowly through a TRIM5α-independent process that is accelerated when capsid-cyclophilin A interactions are inhibited, an effect that may reflect changes in the intrinsic stability of the capsid. Blocking the onset or delaying reverse transcription does not, however, increase viral sensitivity to hTRIM5α, indicating that the recognition of the capsids by hTRIM5α is completed rapidly following entry into the cytoplasm, as previously observed for the simian restriction factors TRIM-Cyp and rhesus TRIM5α.

## Introduction

Following fusion of the HIV-1 envelope with the target-cell membrane, the capsid structure, assembled as a lattice of capsid protein (CA) hexamers and pentamers, and containing the entire replicative machinery of the virus, is released into the cytoplasm [1]. Two important functions of the capsid have been identified. An intact capsid is required to complete at least the initial steps of reverse transcription [2]–[5]. In addition, the capsid appears to participate in intracellular transport of the viral genome to the nucleus through interactions with the cytoskeletal proteins [6].

Although the capsid must eventually be disassembled to permit nuclear transport and integration of the newly synthesized double-stranded DNA, uncertainty has persisted concerning the kinetics of this uncoating process [7], [8]. Several lines of evidence indicate, however, that the uncoating does not occur immediately after entry into the cytoplasm, including the findings that mutations in CA that impair capsid stability lead to a block in viral replication occurring prior to or during reverse transcription [2]–[4], and that one hour after infection, CA can be detected by immunofluorence techniques on a substantial portion of viral particles that enter the cytoplasm by fusion [8]. Importantly, recent studies by Hulme et al [8] indicate that some aspect of reverse transcription influences uncoating, and that inhibiting reverse transcription delays uncoating.

The HIV-1 capsid is also the target of the human restriction factor TRIM5α (hTRIM5α) [9]–[11]. TRIM5α interacts with the mature capsid lattice, not CA monomers, and can directly promote rapid disassembly of the capsid structure, thereby interrupting reverse transcription [12], [13]. TRIM5α possesses an E3 ubiquitin ligase activity that is stimulated following interaction of TRIM5α with the capsid, thereby activating a cascade of events that both promotes innate immune signaling and contributes directly to viral restriction by TRIM5α [14], [15]. HIV-1 carrying the capsid sequence from laboratory-adapted strains (NL4-3, HXB2) and many clinical isolates are poorly recognized by hTRIM5α, and the infectivity of these viruses is inhibited only ≈2-fold in cells expressing physiological levels of hTRIM5α [16]–[20]. We have shown, however, that mutations in CA selected in response to selective pressure exerted by cytotoxic T-lymphocytes in some clinical isolates can increase their sensitivity to hTRIM5α [16], [21].

Although hTRIM5α is known to exert its effects early in the HIV-1 replicative cycle, the kinetics of the interaction between hTRIM5α and the capsid are not well defined. The inhibition of HIV-1 replication by TRIM5-Cyp fusion proteins expressed by some simian species occurs rapidly following entry of the capsid into the cytoplasm [22]–[24], but these fusion proteins recognize the capsid by a mechanism that is distinct from that of TRIM5α, which may influence the kinetics of the interaction [22]. Similarly, rhesus TRIM5α profoundly inhibits HIV-1 replication, but rhesus TRIM5α has a high affinity for the capsid, which may permit rapid binding of a sufficient number of molecules to disrupt the capsid even if maximal binding is not achieved [10], [25], [26]. In contrast, hTRIM5α has a lower affinity for the HIV-1 capsid, which could retard the accumulation of sufficient molecules to exert anti-viral activity [27], [28]. In view of the finding that viral uncoating is linked to reverse transcription, modifications that delay this process would lead to the persistence in the cytoplasm of capsids susceptible to targeting by hTRIM5α. It remains unclear, however, if increasing the time available for capsid-hTRIM5α interactions would actually render viruses more sensitive to hTRIM5α.

To evaluate this question, we have compared the infectivity of viruses with defects known to delay reverse transcription in target cells that express hTRIM5α activity and those in which hTRIM5α activity was inhibited. In addition we have evaluated the impact of delaying the onset of reverse transcription by treatment with a non-nucleoside reverse transcriptase (RT) inhibitor on the sensitivity of HIV-1 to hTRIM5α and capsid stability using viruses with different degrees of susceptibility to this restriction factor. The findings indicate that capsids are rapidly targeted by hTRIM5α, and increasing the time that capsids remain in the cytoplasm does not render the viruses more sensitive to hTRIM5α.

## Methods

### Cell culture

The feline CRFK cell line was obtained from ATCC (Manassas, VA). CRFK cells expressing hTRIM5α, N-terminal HA-tagged hTRIM5α and β-galactosidase were established by transduction with pLenti6/V5-D-TOPO-based vectors as previously described [16]. U373-X4 cells were derived from the human glioblastoma cell line U373-MG as previously described [29]. U373-X4 cells in which hTRIM5α activity has been inhibited by stable overexpression of untagged human TRIM5γ [10], [30]–[33] and the corresponding control cell line that overexpresses β-galactosidase were established by transduction with pLenti6/V5-D-TOPO-based vectors as previously described [16]. All cell lines were maintained in Dulbecco's modified Eagle's medium supplemented with 10% fetal calf serum, 100 U/ml penicillin G and 100 µg/ml streptomycin (complete medium). For U373-X4 cells, the medium also contained 10 µg/ml puromycin and 100 µg/ml hygromycin B. Transduced cells were maintained in medium containing 5 µg/ml (CRFK cells) or 8 µg/ml (U373-X4 cells) blasticidin. Antibiotics other than penicillin G and streptomycin were not used during infectivity assays.

### Production of recombinant viruses

The production of vesicular stomatitis virus (VSV)-pseudotyped pNL4-3-based recombinant viruses that contain a deletion in *env*, that express *Renilla* luciferase in place of Nef, and whose Gag-PR sequences were derived from clinical isolates (NRC2, NRC3, NRC10) or from NL4-3 has been described previously [16], [34]. The recombinant NL4-3-based provirus carrying the RT sequence from a clinical isolate BV34 (accession number JQ994264), which contains numerous mutations associated with resistance to both nucleoside and non-nucleoside RT inhibitors, has been previously described [35]. To transfer this RT sequence to a luciferase-expressing provirus, this plasmid was digested with ClaI and SnaBI, and the fragment was ligated into pNL4-3-ΔENV-lucR-XC [34] cleaved with the same enzymes.

The parental pLAI3 proviral plasmid and variants in which mutations that either disrupt the function of the central polypurine tract (cPPT) and introduce the K188R mutation in integrase (pcPPT-D) or introduce only the K188R mutation without disrupting cPPT function (pcPPT-AG) have previously been described [36], [37] To transfer the sequences encompassing the cPPT to luciferase-expressing proviruses, the following strategy was used. The pBluescript plasmid in which the upstream BssHII site in the polylinker had been mutated, and into which the BssHII-ClaI fragment from NL4-3 had been inserted has previously been described [21]. The SphI-SalI fragment from this plasmid was removed, and replaced with the SphI-SalI fragments from NL-43 (4342 bp) or from pLAI3, pcPPT-D, and pcPPT-AG (4378 bp). A unique PacI restriction site was created in each of the 4 plasmids by introducing into the RNaseH coding sequence upstream of the cPPT a silent mutation (I86, ATA→ATT) by site-directed mutagenesis, using the oligonucleotides described in Table 1. The pNL4-3-based provirus that contains a deletion in *env* and expresses *Renilla* luciferase in place of Nef (pNL4-3-ΔENV-lucR-XC) has previously been described [34]. The SphI-SalI fragment from this plasmid was removed, and replaced by the SphI-SalI fragment containing the PacI restriction site from the NL4-3 pBluescript plasmid, creating pNL4-3-ΔENV-lucR-XC-PacI. Finally, the PacI-SalI fragment from this plasmid was removed, and replaced by the PacI-SalI fragments from each of the three pBluescript plasmids containing sequences from pLAI3, pcPPT-D, and pcPPT-AG, thereby creating pNL4-3-ΔENV-lucR-XC-Bru, pNL4-3-ΔENV-lucR-XC-Bru-D, and pNL4-3-ΔENV-lucR-XC-Bru-AG, respectively. The insert in all of these plasmids was verified by sequencing. VSV-pseudotyped viral stocks were produced as previously described and either used fresh or stored as aliquots at −80°C [16], [34].

**Table 1:** Mutagenesis Primers.

Target	Mutagenesis Primer*
NL4-3	5′ GAGCAGTTAAT**T**AAAAAGGAAAAAGTCTACCTGGCATGGG
LAI3+mutants	5′ GAGCAGTTAAT**T**AAAAAGGAAAAGGTCTATCTGGCATGGG

* Reverse primers were the reverse-complement of the indicated sequence. **T** = mutation introduced.

### Infectivity assays

To measure viral infectivity, CRFK, CRFK-LacZ, CRFK-HA-TRIM5α, and CRFK-TRIM5α cells were plated at 1×10^4^ cells/well in 96-well flat-bottomed plates in 200 µl of complete medium. Twenty-four h later, medium was removed and cells were infected in triplicate with three concentrations of virus (5, 2.5 and 1.25 ng p24/ml) in 200 µl complete medium containing 2 µg/ml DEAE-Dextran. Luciferase activity (relative light units, RLU) was measured as previously described [16], [34] using reagents in the Renilla Luciferase kit (Promega, Madison, WI) and a Varioskan Flash reader (Thermo Fisher Scientific, Waltham, MA). The results were plotted as a function of the amount of virus, and the slope (RLU/ng p24) was determined by linear regression.

To evaluate the effect of inhibiting reverse transcription on sensitivity to TRIM5α, the following protocol was used: 24 h before infection U373-X4, U373-X4-LacZ and U373-X4-TRIM5γ cells were plated at 2×10^4^ cells/well in 96-well flat-bottomed plates in 100 µl of complete medium. Sixteen h before infection, 100 µl of complete medium containing 200 U/ml interferon alpha (IFNα, Sigma-Aldrich, #I4784) was added. On the day of infection, medium was removed and replaced with 100 µl complete medium containing freshly harvested viral supernatants (3 ng p24/well), with or without 250 ng/ml nevirapine (NVP, AIDS Research and Reference Reagent Program). The plates were centrifuged at 260× g for 2 h at 25°C, and transferred to a 37°C/5% CO_2_ incubator. T_0_ was set as the initiation of incubation at 37°C. After 30 min, residual virus was removed by aspirating the medium, washing once with 100 µl of medium of the same composition, and adding 100 µl of medium of the same composition. At various times after infection (1, 2 and 4 h), NVP was removed by aspirating the medium, adding 300 µl of complete medium without NVP, incubating the plates for 10 min at 37°C, aspirating the wash medium, and adding 200 µl of complete medium without NVP. Infection was allowed to proceed for 40 h, after which luciferase activity was measured as described above. In each experiment, all infections were performed in parallel in triplicate wells, and the mean RLU values were used for calculations. In preliminary experiments, we found that incubation of target cells in the continuous presence of 250 ng/ml NVP completely inhibited the infectivity of the recombinant viruses, but that infectivity was restored when NVP was removed using the washing protocol described above (additional file 1, Figure S1).

To evaluate the effect of inhibiting reverse transcription on sensitivity to TRIM5α in cells in which CA-CypA interactions were inhibited, the protocol described above was used, except that all media used for infection, washing and culture contained 1 µg/ml Debio-025 (kindly provided by Debiopharma, Lausanne, Switzerland) [38].

### Statistical analysis

All results are presented as mean ± SEM unless otherwise indicated. Comparisons among groups were performed using ANOVA, followed by the Bonferroni's multiple comparison post-test. To compare the residual infectivity of the different viruses after 4 hours of exposure of target cells to NVP, results from the three cell types were pooled before analysis by ANOVA, followed by Dunnett's multiple comparison test.

## Results

### Impact of delays in reverse transcription on sensitivity to hTRIM5α

**Figure 1:**
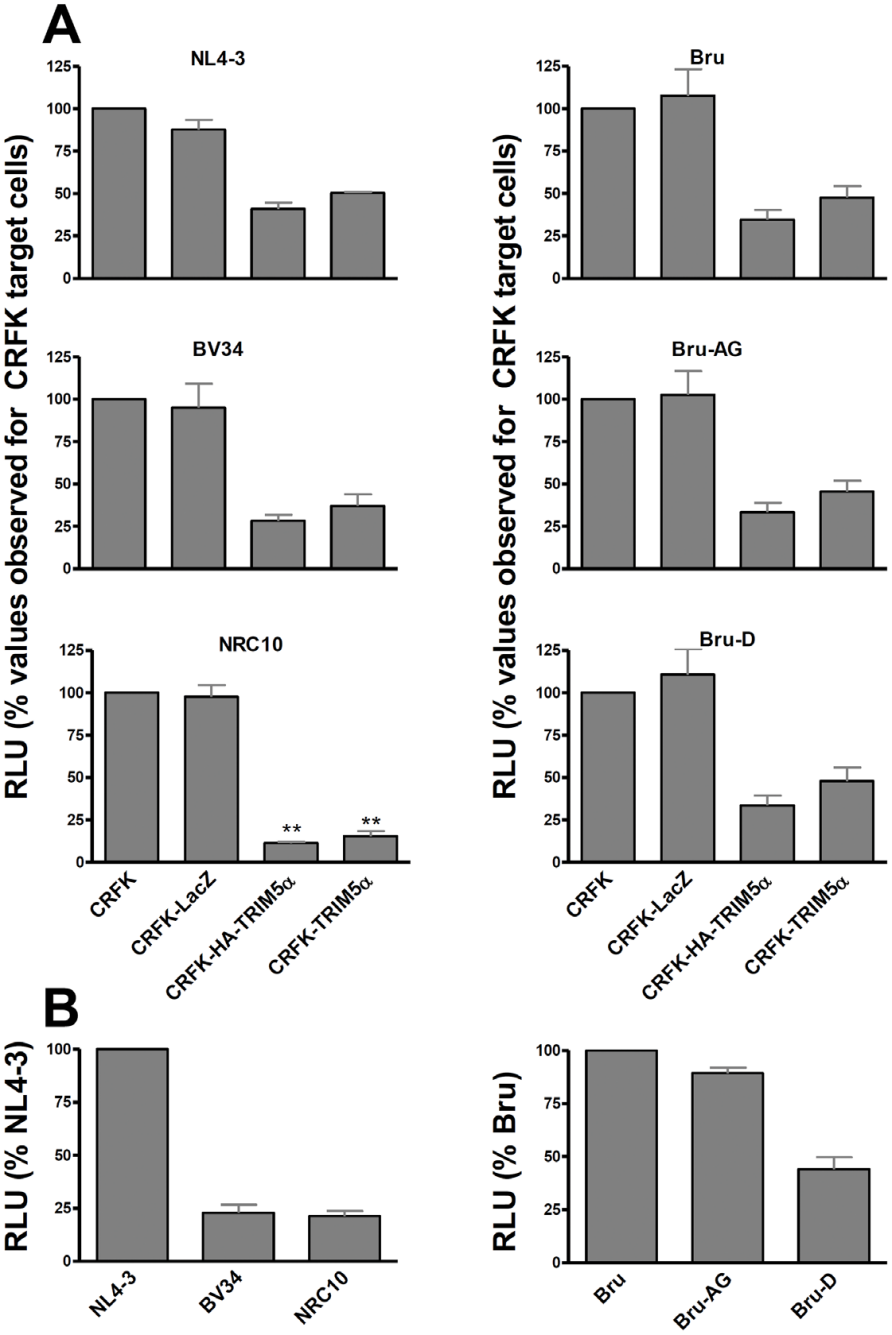
Mutations that delay completion of reverse transcription do not increase viral sensitivity to hTRIM5α. Untransduced CRFK cells, and those transduced with lentiviral vectors resulting in the overexpression of β-galactosidase (CRFK-LacZ), N-terminal hemagglutinin-tagged hTRIM5α (CRFK-HA-TRIM5α) or hTRIM5α were infected with serial two-fold dilutions of the indicated VSV-pseudotyped viruses, which express *Renilla* luciferase in the place of Nef, and RLU was measured 40 h after infection. Infectivity was expressed as the slope of the RLU vs ng p24 curves, determined by linear regression. In the top panels (A), results are the mean ± SEM for three independent experiments expressed relative to infectivity observed in untransduced CRFK cells. ** indicates p<0.01 compared to results for NL4-3 in the same cell line. In the bottom panels (B), results in untransduced CRFK cells for each virus (n = 3) are expressed relative to infectivity observed for NL4-3 (left bottom panel) or Bru (right bottom panel).

Delaying the progression of reverse transcription has been found to slow uncoating of the viral capsid, which could increase the time available for interaction between CA and TRIM5α [8]. To evaluate whether this might increase TRIM5α restriction, we compared the infectivity of viruses expressing the same capsid sequence, but RT proteins with different processivity, in feline CRFK cells and CRFK cells expressing exogenous human TRIM5α. Consistent with prior results, infectivity of the NL4-3 isolate in CRFK cells expressing hTRIM5α was reduced to 45.7±6.6% of that observed in untransduced cells (Figure 1A, left panels). The recombinant virus BV34 carries the RT sequence from a clinical isolate carrying numerous mutations mediating HIV resistance to both nucleoside analogues and non-nucleoside RT inhibitors. Our laboratory previously showed that these mutations delay the completion of reverse transcription by many hours [35], a finding that was confirmed when CRFK cells served as target cells (additional file 1, Figure S2). In TRIM5α-expressing CRFK cells, the infectivity of the BV34 isolate was 32.7±6.9% of that measured in untransduced cells, results not significantly different from those observed for NL4-3, and implying that delayed reverse transcription did not increase susceptibility to hTRIM5α. To make sure that our experimental system was appropriate for demonstrating increased hTRIM5α susceptibility, we tested the recombinant virus NRC10, which carries a CA sequence from a clinical isolate that we have previously shown to be more sensitive to TRIM5α than NL4-3 [16], [21]. In hTRIM5α-expressing CRFK cells, infectivity of NRC10 was reduced to 13.3±4.2% of that observed in untransduced CRFK cells (p<0.001 compared to NL4-3 and BV34). Of note, the infectivity of the BV34 and NRC10 viruses in untransduced CRFK cells were reduced to a similar extent compared to that of NL4-3 (Figure 1B).

Using this system, we also evaluated the effect of delays in plus-strand DNA synthesis on TRIM5α sensitivity. The virus Bru-D has 10 substitutions within the 19 nucleotide long central polypurine tract (cPPT) that prevent priming from the cPPT [37]; these changes also introduce a single amino acid change (K188R in integrase). Preventing priming from the cPPT has been shown to delay the synthesis of plus-strand DNA downstream of the PPT by approximately 1 hour [39]; a delay of similar magnitude was also seen when CRFK cells were used as target cells (additional file 1, Figure S2). The infectivity of the Bru-D virus in cells expressing hTRIM5α was reduced to 40.8±13.4% that observed in untransduced CRFK cells (Figure 1A, right panels); this reduction in infectivity in cells expressing hTRIM5α was not significantly different than that observed for the wild-type Bru (41.2±12.0%) or Bru-AG, a variant that expresses the K188R mutation but has an intact cPPT (39.5±11.4%). Consistent with prior results [36], [37], interrupting the cPTT (Bru-D), but not inserting the K188R mutation alone (Bru-AG), impaired viral infectivity in CRFK cells relative to that of the parental Bru strain (Figure 1B, right panel). Thus, defects that delayed reverse transcription and impaired viral infectivity by two distinct mechanisms (resistance mutations in RT or lack of a cPPT) did not increase sensitivity to hTRIM5α.

### Effect of delaying the onset of reverse transcription on sensitivity to hTRIM5α

We also evaluated the effect of delaying the initiation of HIV-1 reverse transcription on viral sensitivity to hTRIM5α. In these studies we compared results in cells expressing hTRIM5α activity [untransduced U373-X4 cells and U373-X4 cells transduced with a vector resulting in overexpression of β-galactocidase (U373-X4-LacZ)], which both express hTRIM5α, and cells in which hTRIM5α activity had been inhibited by transduction with a vector overexpressing hTRIM5γ (U373-X4-TRIM5γ). Cells were infected with vesicular stomatitis virus (VSV)-G-pseudotyped, NL4-3-based, recombinant viruses containing a deletion in *env* and expressing *Renilla* luciferase in the place of Nef, and in which the Gag-protease (Gag-PR) sequences were derived either from NL4-3 or from clinical isolates (NRC3, NRC2, NRC10). Each cell line was infected with each virus by spinoculation in the presence or absence of 250 ng/ml nevirapine (NVP), and cultured at 37°C for 30 min to permit viral entry. Cells were then washed with the same medium (with or without NVP) to remove residual virus. After varying times of culture, NVP was removed from NVP-treated cultures by aspirating the medium and washing the cells. Medium not containing NVP was then added, and the infection was allowed to proceed for 40 h, after which cell-associated luciferase activity was measured.

**Figure 2:**
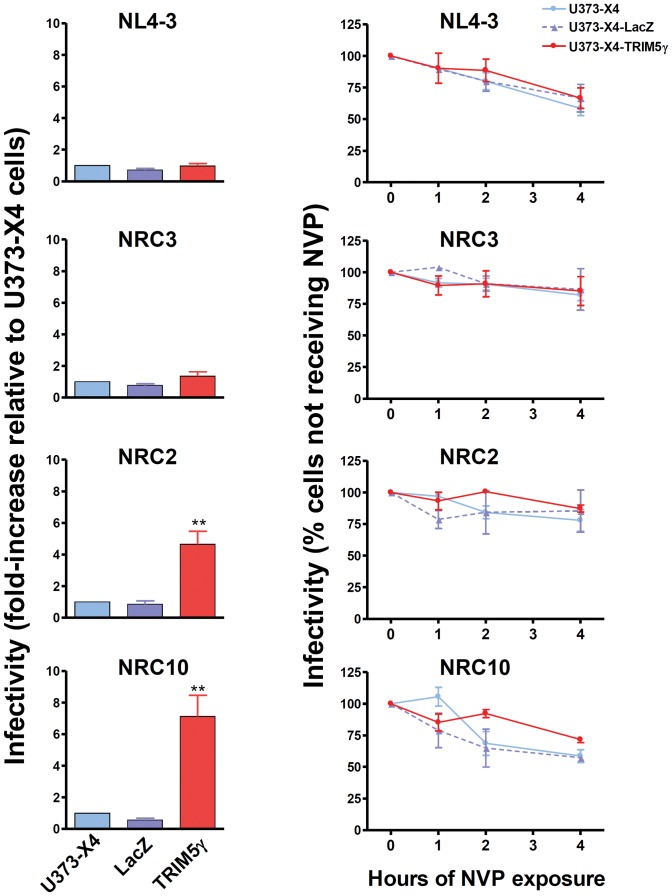
Delaying the onset of reverse transcription does not increase viral sensitivity to hTRIM5α. Untransduced U373-X4 cells and U373-X4 cells transduced with lentiviral vectors resulting in the overexpression of β-galactosidase (LacZ) or human TRIM5γ (TRIM5γ) were cultured overnight in the presence of 100 U/ml IFNα, and infected with 3 ng p24/well of the indicated recombinant VSV-pseudotyped viruses, which express *Renilla* luciferase in the place of Nef, and luciferase activity was measured 40 h after infection. Parallel cultures were maintained in the presence of 250 ng/ml NVP for the indicated times prior to washing the cells to remove NVP. Cultures not receiving NVP were washed 1 h after infection. In the left panels, results for cells not treated with NVP are expressed relative to RLU measured in untransduced U373-X4 cells. In the right panels, results for each cell line are expressed relative to RLU measured in cultures not treated with NVP. Shown are the mean ± SEM for three independent experiments performed using fresh viral stocks. ** indicates p<0.01 compared to U373-X4-LacZ cells.

As previously described [16], the infectivity of viruses carrying the CA sequence from NL4-3 and clinical isolate NRC3 were similar in cells in which hTRIM5α activity had or had not been inhibited by overexpression of TRIM5γ (Figure 2, left panels). In contrast, the infectivity of viruses carrying the CA sequence from clinical isolates NRC2 and NRC10 was increased 5-fold and 8-fold, respectively, in cells in which hTRIM5α activity had been inhibited by overexpression of TRIM5γ, indicating that these viruses were substantially more sensitive to restriction by the levels of hTRIM5α activity expressed in U373-X4 cells.

Delaying the onset of reverse transcription by NVP treatment led to a time-dependent decrease in infectivity for all viruses studied (Figure 2, right panels). For all viruses studied, however, no differences in the loss of infectivity were observed comparing the infection of cells in which hTRIM5α activity had or had not been inhibited by overexpression of TRIM5γ. This was true both for viruses that were resistant (NL4-3, NRC3) or sensitive to hTRIM5α (NRC2, NRC10). After delaying reverse transcription by 4 hours, the residual infectivity of NL4-3 viruses in the target cells had decreased to 61±16% of values observed when infection was allowed to proceed without interruption. This residual infectivity was significantly less than that seen for NRC3 (84±14%, p<0.05) and NRC2 (84±17%, p<0.05), but similar to that observed for NRC10 (60±8%). Thus, the loss of infectivity resulting from a delay in the onset of reverse transcription, likely to reflect intrinsic capsid stability, seemed to be virus-dependent, but did not correlate with sensitivity to hTRIM5α, and delaying the onset of reverse transcription for several hours did not result in an increased hTRIM5α-dependent loss in infectivity.

### Effect of delaying the onset of reverse transcription on sensitivity to hTRIM5α after inhibiting cyclophilin A-CA interactions

The inhibition of cyclophilin A (CypA)-CA interactions by treatment of cells with cyclosporin A or the nonimmunosuppressive cyclosporin A analog Debio-025 has been shown to impair HIV-1 replication [40]–[42], reduce capsid stability [4], and can also increase or decrease the sensitivity of HIV-1 to hTRIM5α in a capsid-specific fashion [16]. Thus, we also evaluated whether inhibiting CypA-CA interactions would affect viral sensitivity to hTRIM5α when the onset of reverse transcription was delayed. To do so, we repeated the NVP time-course experiments using target cells treated with 1 µM Debio-025.

**Figure 3:**
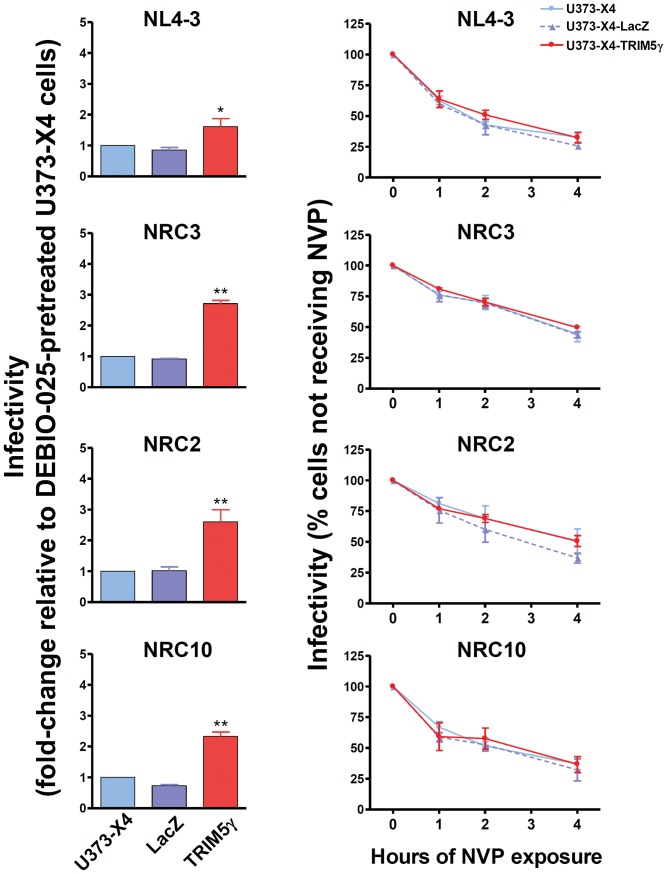
Delaying the onset of reverse transcription under conditions where CypA-CA interactions are inhibited does not increase viral sensitivity to hTRIM5α. Experiments were performed and results are expressed as in Figure 2, legend, except that all cultures were maintained in the continuous presence of 1 µg/ml Debio-025. Shown are the mean ± SEM for three independent experiments performed using fresh viral stocks. * p<0.05, ** p<0.01 compared to U373-X4-LacZ cells.

As previously reported [16], following the inhibition of CypA-CA interactions, viruses carrying the NL4-3 and NRC3 capsids showed modest sensitivity to hTRIM5α. NRC2 and NRC10 remained sensitive to hTRIM5α, but the sensitivity of NRC10 to hTRIM5α was significantly lower in Debio-025 treated cells than in untreated cells (p<0.05, compare left panels in Figures 2 and 3).

Delaying the onset of reverse transcription by NVP treatment led to a time-dependent decrease in infectivity for all viruses (Figure 3, right panels), but as for target cells not treated with Debio-025, no differences in the loss of infectivity were observed comparing the infection of cells in which hTRIM5α activity had or had not been inhibited by overexpression of TRIM5γ. Delaying the onset of reverse transcription led to a more rapid loss in viral infectivity when CypA-CA interactions were inhibited (p<0.001 for all viruses, comparing residual infectivity after 4 hr of NVP treatment in untreated and Debio-025 treated cells). As was seen in cells not treated with Debio-025, the residual infectivity of NL4-3 virus after 4 hr of NVP treatment (30±2%) was significantly less than that of NRC3 (46±2%, p<0.01) and NRC2 (46±4%, p<0.01), but not that observed for NRC10 (35±4%). Thus, inhibiting CypA-CA interactions appeared to impair capsid stability and could modify viral sensitivity to hTRIM5α, but did not result in increased hTRIM5α-dependent loss in infectivity when the onset of reverse transcription was delayed for several hours.

## Discussion

To explore the kinetics of the recognition of the HIV-1 capsid by hTRIM5α, we evaluated the effect of delaying reverse transcription on viral sensitivity to this restriction factor. Although inhibiting reverse transcription increases the time that an intact capsid can be recognized, we found that this did not increase viral sensitivity to TRIM5α, indicating that the recognition of the capsid by TRIM5α must occur rapidly after entry of the capsid into the cytoplasm, and is not facilitated by delaying uncoating.

Recent work strongly supports the conclusion that reverse transcription facilitates uncoating [8]. Although uncoating ultimately renders the preintegration complex resistant to TRIM5α, it is unclear how much CA must be removed to achieve this result [8]. It is also conceivable that the initial stages of uncoating could increase the ability TRIM5α to recognize or destabilize the capsid. If this were true, the failure of NVP treatment to increase sensitivity to TRIM5α might be attributable, at least in part, to the failure of reverse transcription to induce this hypothetical TRIM5α-sensitive state. In this regard, our experiments evaluating viruses in which reverse transcription is delayed but not prevented are important. Viruses carrying mutations in RT (BV34) or in the cPPT (Bru-D), which delay DNA synthesis throughout the process of reverse transcription or during the synthesis of plus-strand DNA, respectively, did not display increased sensitivity to hTRIM5α, arguing against a transient period of increased sensitivity occurring during reverse transcription. In these studies, hTRIM5α activity was expressed in feline CRFK cells, and cell line-specific effects on the expression of TRIM5a activity have been reported [43]. Similar results for the BV34 virus were seen using human U373-X4-TRIM5γ and U373-X4-lacZ cell lines (data not shown).

The mechanism(s) of action of TRIM5α are not completely defined. Current evidence suggests that the E3 activity of TRIM5α contributes to the block in viral replication occurring early in reverse transcription [12], [14], [15], [33], [44]–[46], and is likely to involve proteasome-mediated degradation [44], [47], [48]. The importance of both the E3 activity of TRIM5α and proteasomal degradation in viral restriction, however, appears to depend on both the TRIM5α protein used and the restricted virus [10], [13], [14], [17], [44]–[49], and TRIM5α-induced blocks occurring before and after the completion of reverse transcription have been described [48], [50]–[52]. Our findings indicate, however, that regardless of the pathway and kinetics of viral destruction, the recognition phase of viral capsids whose infectivity will ultimately be inhibited by TRIM5α is accomplished rapidly after their entry into the cytoplasm.

Our findings also confirm i) our previous observation that CA-CypA interactions can increase or decrease sensitivity to hTRIM5α in a strain-specific fashion [16], and ii) studies from several groups demonstrating that CypA binding also improves the stability of the HIV-1 capsid in a hTRIM5α-independent fashion following its release into the cytoplasm [4], [17], [19], [20], [40]–[42], [53]. For the viruses studied by us, CypA binding appeared to improve capsid stability to a similar extent, despite that these viruses displayed variable sensitivity to hTRIM5α. HIV-1 carrying capsid sequences whose stability is impaired by CypA have also been described [4], but none of our viruses had this phenotype.

### Conclusions

Consistent with prior studies, our findings indicate that following entry of the HIV-1 capsid into the cytoplasm, two distinct processes can lead to a loss in viral infectivity. First, the capsid can be targeted for destruction by hTRIM5α to an extent that depends on its sensitivity to this restriction factor. Second, capsids can be lost more slowly through a hTRIM5α-independent process that is accelerated when CA-CypA interactions are inhibited, an effect that may reflect changes in the intrinsic stability of the capsid. Blocking the onset or delaying reverse transcription does not increase viral sensitivity to hTRIM5α, indicating that the recognition of the capsids by hTRIM5α is completed rapidly following entry into the cytoplasm, as previously observed for the simian restriction factors TRIM-Cyp and rhesus TRIM5α.

## Supporting Information

Figure S1 **Reversibility of the inhibition of reverse transcription by nevirapine.** U373-X4 cells were plated at 2×10^4^ cells/well in 96-well flat-bottomed plates in 100 µl of complete medium. Sixteen h before infection, 100 µl of complete medium containing 200 U/ml interferon alpha was added. On the day of infection, medium was removed and replaced with 100 µl complete medium containing NL4-3 (3 ng p24/well) and the indicated concentrations of NVP. The plates were centrifuged at 260× g for 2 h at 25°C, and transferred to a 37°C/5% CO_2_ incubator. T_0_ was set as the initiation of incubation at 37°C. After 30 min, residual virus was removed by aspirating the medium, washing once with 100 µl of medium of the same composition, and adding 100 µl of medium of the same composition. At 2 h, NVP wells were washed using the procedure described in the Materials and Methods using medium containing the original concentration of NVP or no NVP. Infection was allowed to proceed for 40 h, after which luciferase activity was measured. Results are the mean ± SEM for triplicate determinations from one of two experiments that gave similar results.
(TIF)

Figure S2 **Effect of mutations in reverse transcriptase or the cPPT on the kinetics of reverse transcription.** CRFK cells transduced with lentiviral vectors resulting in the overexpression of β-galactosidase (CRFK-LacZ) were plated at 1×10^5^ cells/well in 96-well plates in100 µl of complete medium. Twenty-four h later, 50 µl of medium was added containing 50 ng p24/ml of the indicated VSV-pseudotyped viruses, which express *Renilla* luciferase in the place of Nef. The plates were centrifuged (300×g; 2 h, 32°C), after which the supernatant was removed and replaced with 150 µl complete medium, and the plates were incubated at 37°C (t = zero). At the indicated times, 50 µl of medium containing 800 µM 3TC (A) or 1 µg/ml NVP (B) was added to triplicate wells. Luciferase activity (RLU) was measured 40 h after infection. Results are expressed as the percentage of values obtained for cells treated with RT inhibitors 24 h after infection, and are the mean ± SEM for 2 (panel A) or 4 (panel B) independent experiments. * indicates p<0.02.
(TIF)

## References

[1] Ganser-Pornillos BK, Yeager M, Sundquist WI (2008) The structural biology of HIV assembly. Curr Opin Struct Biol 18: 203–217. 18406133 10.1016/j.sbi.2008.02.001 PMC2819415

[2] Forshey BM, von Schwedler U, Sundquist WI, Aiken C (2002) Formation of a human immunodeficiency virus type 1 core of optimal stability is crucial for viral replication. J Virol 76: 5667–5677. 11991995 10.1128/JVI.76.11.5667-5677.2002 PMC137032

[3] Leschonsky B, Ludwig C, Bieler K, Wagner R (2007) Capsid stability and replication of human immunodeficiency virus type 1 are influenced critically by charge and size of Gag residue 183. J Gen Virol 88: 207–216. 17170453 10.1099/vir.0.81894-0

[4] Li Y, Kar AK, Sodroski J (2009) Target cell type-dependent modulation of human immunodeficiency virus type 1 capsid disassembly by cyclophilin A. J Virol 83: 10951–10962. 19656870 10.1128/JVI.00682-09 PMC2772774

[5] Arhel NJ, Souquere-Besse S, Munier S, Souque P, Guadagnini S, et al (2007) HIV-1 DNA Flap formation promotes uncoating of the pre-integration complex at the nuclear pore. EMBO J 26: 3025–3037. 17557080 10.1038/sj.emboj.7601740 PMC1894778

[6] McDonald D, Vodicka MA, Lucero G, Svitkina TM, Borisy GG, et al (2002) Visualization of the intracellular behavior of HIV in living cells. J Cell Biol 159: 441–452. 12417576 10.1083/jcb.200203150 PMC2173076

[7] Arhel N (2010) Revisiting HIV-1 uncoating. Retrovirology 7: 96. 21083892 10.1186/1742-4690-7-96 PMC2998454

[8] Hulme AE, Perez O, Hope TJ (2011) Complementary assays reveal a relationship between HIV-1 uncoating and reverse transcription. Proc Natl Acad Sci U S A 108: 9975–9980. 21628558 10.1073/pnas.1014522108 PMC3116424

[9] Luban J (2007) Cyclophilin A, TRIM5, and resistance to human immunodeficiency virus type 1 infection. J Virol 81: 1054–1061. 16956947 10.1128/JVI.01519-06 PMC1797489

[10] Stremlau M, Owens CM, Perron MJ, Kiessling M, Autissier P, et al (2004) The cytoplasmic body component TRIM5alpha restricts HIV-1 infection in Old World monkeys. Nature 427: 848–853. 14985764 10.1038/nature02343

[11] Towers GJ (2007) The control of viral infection by tripartite motif proteins and cyclophilin A. Retrovirology 4: 40. 17565686 10.1186/1742-4690-4-40 PMC1906832

[12] Diaz-Griffero F, Kar A, Perron M, Xiang SH, Javanbakht H, et al (2007) Modulation of retroviral restriction and proteasome inhibitor-resistant turnover by changes in the TRIM5alpha B-box 2 domain. J Virol 81: 10362–10378. 17626085 10.1128/JVI.00703-07 PMC2045480

[13] Stremlau M, Perron M, Lee M, Li Y, Song B, et al (2006) Specific recognition and accelerated uncoating of retroviral capsids by the TRIM5alpha restriction factor. Proc Natl Acad Sci U S A 103: 5514–5519. 16540544 10.1073/pnas.0509996103 PMC1459386

[14] Kim J, Tipper C, Sodroski J (2011) Role of TRIM5alpha RING domain E3 ubiquitin ligase activity in capsid disassembly, reverse transcription blockade, and restriction of simian immunodeficiency virus. J Virol 85: 8116–8132. 21680520 10.1128/JVI.00341-11 PMC3147946

[15] Pertel T, Hausmann S, Morger D, Zuger S, Guerra J, et al (2011) TRIM5 is an innate immune sensor for the retrovirus capsid lattice. Nature 472: 361–365. 21512573 10.1038/nature09976 PMC3081621

[16] Battivelli E, Lecossier D, Matsuoka S, Migraine J, Clavel F, et al (2010) Strain-specific differences in the impact of human TRIM5alpha, different TRIM5alpha alleles, and the inhibition of capsid-cyclophilin A interactions on the infectivity of HIV-1. J Virol 84: 11010–11019. 20702630 10.1128/JVI.00758-10 PMC2953162

[17] Hatziioannou T, Perez-Caballero D, Cowan S, Bieniasz PD (2005) Cyclophilin interactions with incoming human immunodeficiency virus type 1 capsids with opposing effects on infectivity in human cells. J Virol 79: 176–183. 15596813 10.1128/JVI.79.1.176-183.2005 PMC538701

[18] Keckesova Z, Ylinen LM, Towers GJ (2006) Cyclophilin A renders human immunodeficiency virus type 1 sensitive to Old World monkey but not human TRIM5 alpha antiviral activity. J Virol 80: 4683–4690. 16641261 10.1128/JVI.80.10.4683-4690.2006 PMC1472055

[19] Sokolskaja E, Berthoux L, Luban J (2006) Cyclophilin A and TRIM5alpha independently regulate human immunodeficiency virus type 1 infectivity in human cells. J Virol 80: 2855–2862. 16501094 10.1128/JVI.80.6.2855-2862.2006 PMC1395419

[20] Stremlau M, Song B, Javanbakht H, Perron M, Sodroski J (2006) Cyclophilin A: an auxiliary but not necessary cofactor for TRIM5alpha restriction of HIV-1. Virology 351: 112–120. 16643975 10.1016/j.virol.2006.03.015

[21] Battivelli E, Migraine J, Lecossier D, Yeni P, Clavel F, et al (2011) Gag cytotoxic T lymphocyte escape mutations can increase sensitivity of HIV-1 to human TRIM5alpha, linking intrinsic and acquired immunity. J Virol 85: 11846–11854. 21917976 10.1128/JVI.05201-11 PMC3209307

[22] Perez-Caballero D, Hatziioannou T, Zhang F, Cowan S, Bieniasz PD (2005) Restriction of human immunodeficiency virus type 1 by TRIM-CypA occurs with rapid kinetics and independently of cytoplasmic bodies, ubiquitin, and proteasome activity. J Virol 79: 15567–15572. 16306627 10.1128/JVI.79.24.15567-15572.2005 PMC1316013

[23] Nisole S, Lynch C, Stoye JP, Yap MW (2004) A Trim5-cyclophilin A fusion protein found in owl monkey kidney cells can restrict HIV-1. Proc Natl Acad Sci U S A 101: 13324–13328. 15326303 10.1073/pnas.0404640101 PMC516566

[24] Sayah DM, Sokolskaja E, Berthoux L, Luban J (2004) Cyclophilin A retrotransposition into TRIM5 explains owl monkey resistance to HIV-1. Nature 430: 569–573. 15243629 10.1038/nature02777

[25] Kratovac Z, Virgen CA, Bibollet-Ruche F, Hahn BH, Bieniasz PD, et al (2008) Primate lentivirus capsid sensitivity to TRIM5 proteins. J Virol 82: 6772–6777. 18417575 10.1128/JVI.00410-08 PMC2447065

[26] Campbell EM, Perez O, Anderson JL, Hope TJ (2008) Visualization of a proteasome-independent intermediate during restriction of HIV-1 by rhesus TRIM5alpha. J Cell Biol 180: 549–561. 18250195 10.1083/jcb.200706154 PMC2234241

[27] Ganser-Pornillos BK, Chandrasekaran V, Pornillos O, Sodroski JG, Sundquist WI, et al (2011) Hexagonal assembly of a restricting TRIM5alpha protein. Proc Natl Acad Sci U S A 108: 534–539. 21187419 10.1073/pnas.1013426108 PMC3021009

[28] Luban J (2012) TRIM5 and the Regulation of HIV-1 Infectivity. Mol Biol Int 2012: 426840. 22701176 10.1155/2012/426840 PMC3369500

[29] Labrosse B, Brelot A, Heveker N, Sol N, Schols D, et al (1998) Determinants for sensitivity of human immunodeficiency virus coreceptor CXCR4 to the bicyclam AMD3100. J Virol 72: 6381–6388. 9658078 10.1128/jvi.72.8.6381-6388.1998 PMC109787

[30] Battivelli E, Migraine J, Lecossier D, Matsuoka S, Perez-Bercoff D, et al (2011) Modulation of TRIM5alpha activity in human cells by alternatively spliced TRIM5 isoforms. J Virol 85: 7828–7835. 21632761 10.1128/JVI.00648-11 PMC3147942

[31] Maegawa H, Nakayama EE, Kuroishi A, Shioda T (2008) Silencing of tripartite motif protein (TRIM) 5alpha mediated anti-HIV-1 activity by truncated mutant of TRIM5alpha. J Virol Methods 151: 249–256. 18524394 10.1016/j.jviromet.2008.04.012

[32] Passerini LD, Keckesova Z, Towers GJ (2006) Retroviral restriction factors Fv1 and TRIM5alpha act independently and can compete for incoming virus before reverse transcription. J Virol 80: 2100–2105. 16474118 10.1128/JVI.80.5.2100-2105.2006 PMC1395401

[33] Perez-Caballero D, Hatziioannou T, Yang A, Cowan S, Bieniasz PD (2005) Human tripartite motif 5alpha domains responsible for retrovirus restriction activity and specificity. J Virol 79: 8969–8978. 15994791 10.1128/JVI.79.14.8969-8978.2005 PMC1168745

[34] Matsuoka S, Dam E, Lecossier D, Clavel F, Hance AJ (2009) Modulation of HIV-1 infectivity and cyclophilin A-dependence by Gag sequence and target cell type. Retrovirology 6: 21. 19254360 10.1186/1742-4690-6-21 PMC2653016

[35] Bouchonnet F, Dam E, Mammano F, de Soultrait V, Hennere G, et al (2005) Quantification of the effects on viral DNA synthesis of reverse transcriptase mutations conferring human immunodeficiency virus type 1 resistance to nucleoside analogues. J Virol 79: 812–822. 15613309 10.1128/JVI.79.2.812-822.2005 PMC538537

[36] Charneau P, Alizon M, Clavel F (1992) A second origin of DNA plus-strand synthesis is required for optimal human immunodeficiency virus replication. J Virol 66: 2814–2820. 1560526 10.1128/jvi.66.5.2814-2820.1992 PMC241038

[37] Zennou V, Petit C, Guetard D, Nerhbass U, Montagnier L, et al (2000) HIV-1 genome nuclear import is mediated by a central DNA flap. Cell 101: 173–185. 10786833 10.1016/S0092-8674(00)80828-4

[38] Ptak RG, Gallay PA, Jochmans D, Halestrap AP, Ruegg UT, et al (2008) Inhibition of human immunodeficiency virus type 1 replication in human cells by Debio-025, a novel cyclophilin binding agent. Antimicrob Agents Chemother 52: 1302–1317. 18212100 10.1128/AAC.01324-07 PMC2292519

[39] Thomas DC, Voronin YA, Nikolenko GN, Chen J, Hu WS, et al (2007) Determination of the ex vivo rates of human immunodeficiency virus type 1 reverse transcription by using novel strand-specific amplification analysis. J Virol 81: 4798–4807. 17314159 10.1128/JVI.02471-06 PMC1900155

[40] Billich A, Hammerschmid F, Peichl P, Wenger R, Zenke G, et al (1995) Mode of action of SDZ NIM 811, a nonimmunosuppressive cyclosporin A analog with activity against human immunodeficiency virus (HIV) type 1: interference with HIV protein-cyclophilin A interactions. J Virol 69: 2451–2461. 7884893 10.1128/jvi.69.4.2451-2461.1995 PMC188920

[41] Luban J, Bossolt KL, Franke EK, Kalpana GV, Goff SP (1993) Human immunodeficiency virus type 1 Gag protein binds to cyclophilins A and B. Cell 73: 1067–1078. 8513493 10.1016/0092-8674(93)90637-6

[42] Thali M, Bukovsky A, Kondo E, Rosenwirth B, Walsh CT, et al (1994) Functional association of cyclophilin A with HIV-1 virions. Nature 372: 363–365. 7969495 10.1038/372363a0

[43] Berube J, Bouchard A, Berthoux L (2007) Both TRIM5alpha and TRIMCyp have only weak antiviral activity in canine D17 cells. Retrovirology 4: 68. 17892575 10.1186/1742-4690-4-68 PMC2064933

[44] Diaz-Griffero F, Kar A, Lee M, Stremlau M, Poeschla E, et al (2007) Comparative requirements for the restriction of retrovirus infection by TRIM5alpha and TRIMCyp. Virology 369: 400–410. 17920096 10.1016/j.virol.2007.08.032 PMC2153441

[45] Javanbakht H, Diaz-Griffero F, Stremlau M, Si Z, Sodroski J (2005) The contribution of RING and B-box 2 domains to retroviral restriction mediated by monkey TRIM5alpha. J Biol Chem 280: 26933–26940. 15897199 10.1074/jbc.M502145200

[46] Maegawa H, Miyamoto T, Sakuragi J, Shioda T, Nakayama EE (2010) Contribution of RING domain to retrovirus restriction by TRIM5alpha depends on combination of host and virus. Virology 399: 212–220. 20110098 10.1016/j.virol.2010.01.003

[47] Anderson JL, Campbell EM, Wu X, Vandegraaff N, Engelman A, et al (2006) Proteasome inhibition reveals that a functional preintegration complex intermediate can be generated during restriction by diverse TRIM5 proteins. J Virol 80: 9754–9760. 16973579 10.1128/JVI.01052-06 PMC1617233

[48] Rold CJ, Aiken C (2008) Proteasomal degradation of TRIM5alpha during retrovirus restriction. PLoS Pathog 4: e1000074. 18497858 10.1371/journal.ppat.1000074 PMC2374908

[49] Zhao G, Ke D, Vu T, Ahn J, Shah VB, et al (2011) Rhesus TRIM5alpha disrupts the HIV-1 capsid at the inter-hexamer interfaces. PLoS Pathog 7: e1002009. 21455494 10.1371/journal.ppat.1002009 PMC3063768

[50] Wu X, Anderson JL, Campbell EM, Joseph AM, Hope TJ (2006) Proteasome inhibitors uncouple rhesus TRIM5alpha restriction of HIV-1 reverse transcription and infection. Proc Natl Acad Sci U S A 103: 7465–7470. 16648264 10.1073/pnas.0510483103 PMC1464362

[51] Yap MW, Dodding MP, Stoye JP (2006) Trim-cyclophilin A fusion proteins can restrict human immunodeficiency virus type 1 infection at two distinct phases in the viral life cycle. J Virol 80: 4061–4067. 16571822 10.1128/JVI.80.8.4061-4067.2006 PMC1440439

[52] Ylinen LM, Keckesova Z, Wilson SJ, Ranasinghe S, Towers GJ (2005) Differential restriction of human immunodeficiency virus type 2 and simian immunodeficiency virus SIVmac by TRIM5alpha alleles. J Virol 79: 11580–11587. 16140735 10.1128/JVI.79.18.11580-11587.2005 PMC1212619

[53] Yin L, Braaten D, Luban J (1998) Human immunodeficiency virus type 1 replication is modulated by host cyclophilin A expression levels. J Virol 72: 6430–6436. 9658084 10.1128/jvi.72.8.6430-6436.1998 PMC109799

