# Supplementary figures and images for: Delaying Reverse Transcription Does Not Increase Sensitivity of HIV-1 to Human TRIM5α
Source: PLoS One. 2013 Jan 8;8(1):e52434. doi: 10.1371/journal.pone.0052434 (PMC3540060; doi:10.1371/journal.pone.0052434)

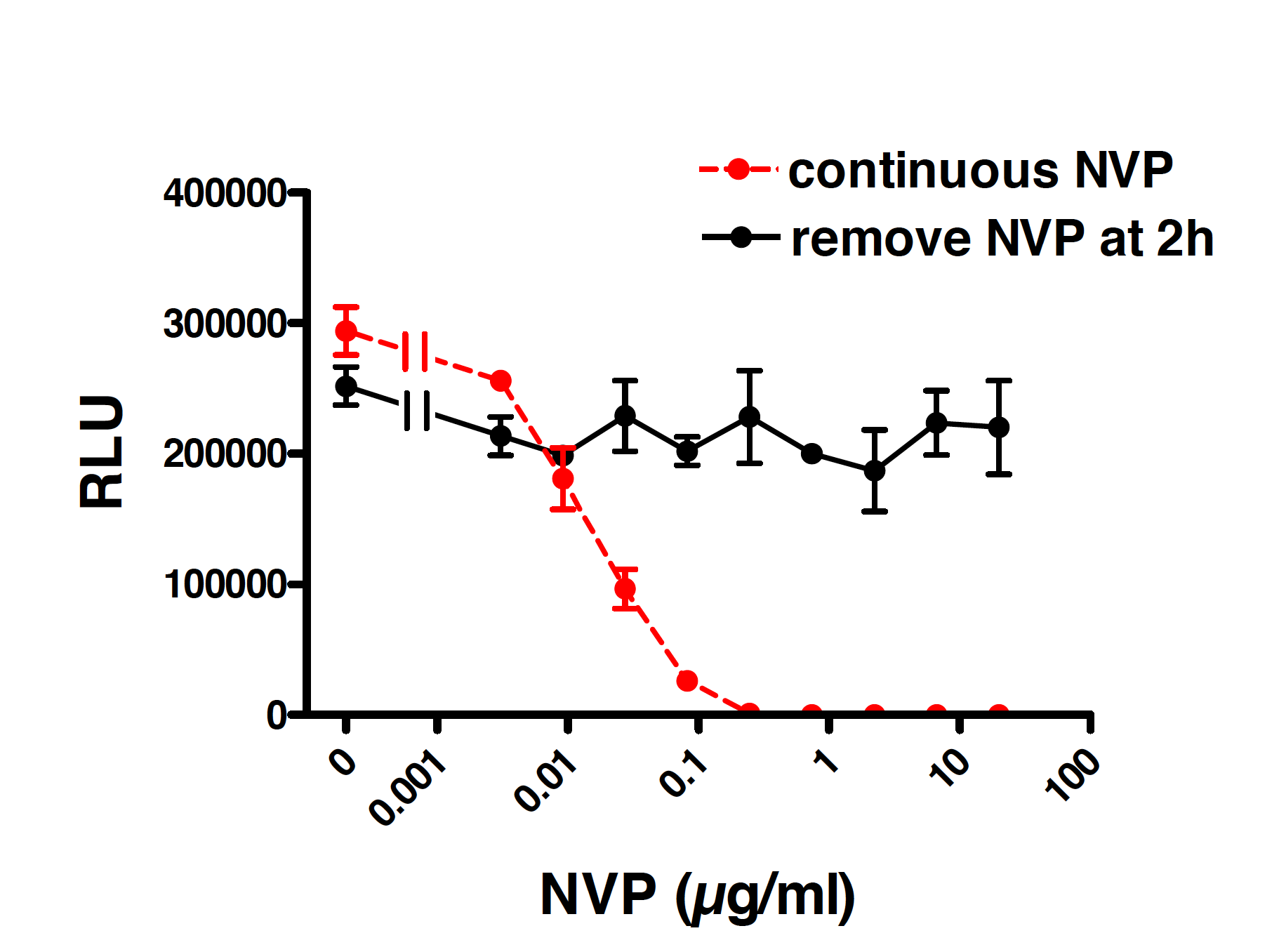

Supplement: Figure S1 — Reversibility of the inhibition of reverse transcription by nevirapine. U373-X4 cells were plated at 2×10^4^ cells/well in 96-well flat-bottomed plates in 100 µl of complete medium. Sixteen h before infection, 100 µl of complete medium containing 200 U/ml interferon alpha was added. On the day of infection, medium was removed and replaced with 100 µl complete medium containing NL4-3 (3 ng p24/well) and the indicated concentrations of NVP. The plates were centrifuged at 260× g for 2 h at 25°C, and transferred to a 37°C/5% CO2 incubator. T0 was set as the initiation of incubation at 37°C. After 30 min, residual virus was removed by aspirating the medium, washing once with 100 µl of medium of the same composition, and adding 100 µl of medium of the same composition. At 2 h, NVP wells were washed using the procedure described in the Materials and Methods using medium containing the original concentration of NVP or no NVP. Infection was allowed to proceed for 40 h, after which luciferase activity was measured. Results are the mean ± SEM for triplicate determinations from one of two experiments that gave similar results. (TIF) [file pone.0052434.s001.tif]

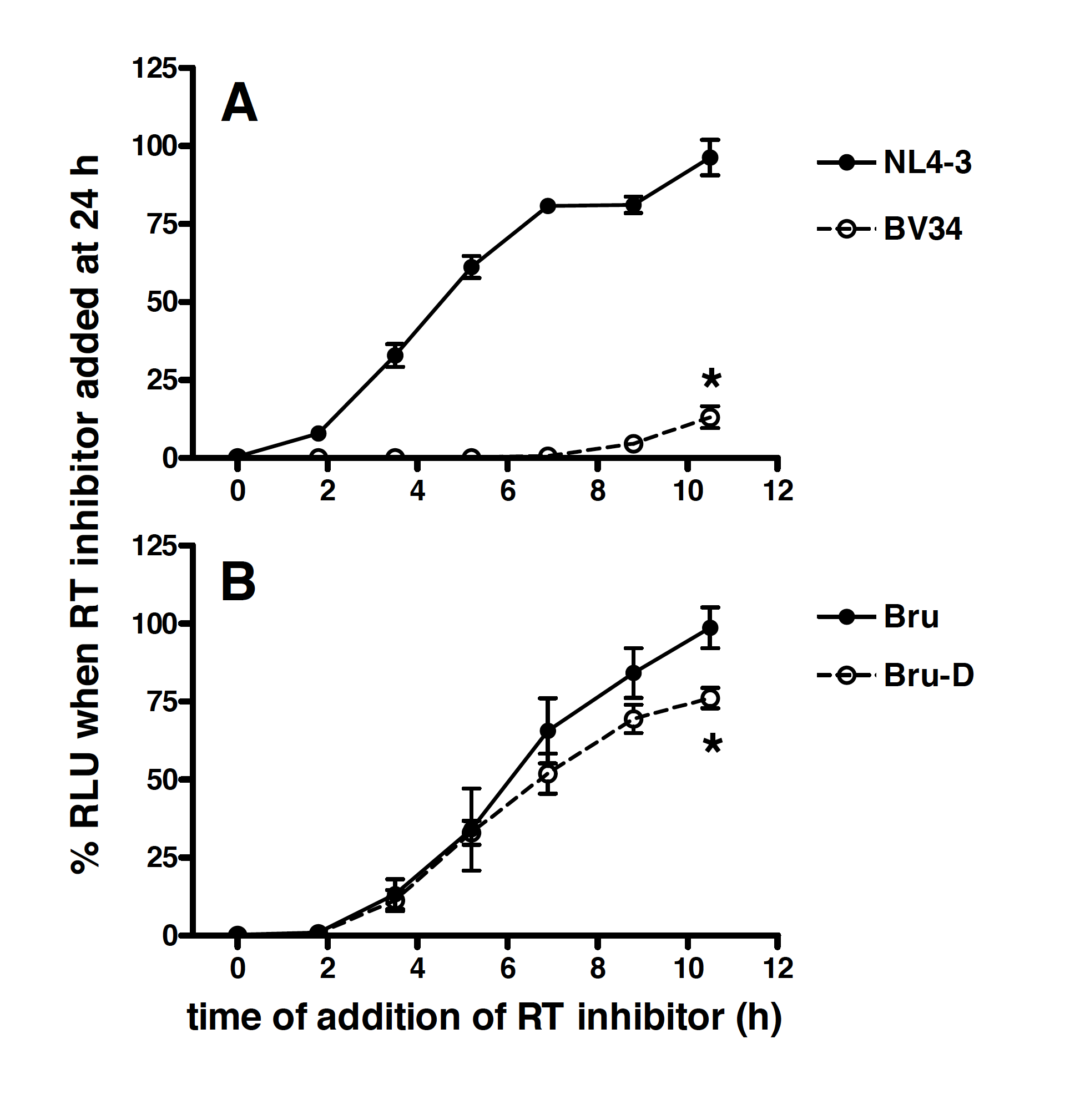

Supplement: Figure S2 — Effect of mutations in reverse transcriptase or the cPPT on the kinetics of reverse transcription. CRFK cells transduced with lentiviral vectors resulting in the overexpression of β-galactosidase (CRFK-LacZ) were plated at 1×10^5^ cells/well in 96-well plates in100 µl of complete medium. Twenty-four h later, 50 µl of medium was added containing 50 ng p24/ml of the indicated VSV-pseudotyped viruses, which express Renilla luciferase in the place of Nef. The plates were centrifuged (300×g; 2 h, 32°C), after which the supernatant was removed and replaced with 150 µl complete medium, and the plates were incubated at 37°C (t = zero). At the indicated times, 50 µl of medium containing 800 µM 3TC (A) or 1 µg/ml NVP (B) was added to triplicate wells. Luciferase activity (RLU) was measured 40 h after infection. Results are expressed as the percentage of values obtained for cells treated with RT inhibitors 24 h after infection, and are the mean ± SEM for 2 (panel A) or 4 (panel B) independent experiments. * indicates p<0.02. (TIF) [file pone.0052434.s002.tif]
